# Neuroserpin: structure, function, physiology and pathology

**DOI:** 10.1007/s00018-021-03907-6

**Published:** 2021-08-17

**Authors:** Emanuela D’Acunto, Annamaria Fra, Cristina Visentin, Mauro Manno, Stefano Ricagno, Giovanna Galliciotti, Elena Miranda

**Affiliations:** 1 Department of Biology and Biotechnologies ‘Charles Darwin’, Sapienza University of Rome, Rome, Italy; 2 Department of Molecular and Translational Medicine, University of Brescia, Brescia, Italy; 3 Department of Biosciences, University of Milan, Milan, Italy; 4 Institute of Molecular and Translational Cardiology, I.R.C.C.S. Policlinico San Donato, Milan, Italy; 5 Institute of Biophysics, National Research Council of Italy, Palermo, Italy; 6 Institute of Neuropathology, University Medical Center Hamburg-Eppendorf, Hamburg, Germany; 7 Pasteur Institute—Cenci Bolognetti Foundation, Sapienza University of Rome, Rome, Italy

**Keywords:** Serpins, Synaptic plasticity, Tissue-type plasminogen activator, Neurodegenerative disease, Epilepsy, Pathogenic variants

## Abstract

Neuroserpin is a serine protease inhibitor identified in a search for proteins implicated in neuronal axon growth and synapse formation. Since its discovery over 30 years ago, it has been the focus of active research. Many efforts have concentrated in elucidating its neuroprotective role in brain ischemic lesions, the structural bases of neuroserpin conformational change and the effects of neuroserpin polymers that underlie the neurodegenerative disease FENIB (familial encephalopathy with neuroserpin inclusion bodies), but the investigation of the physiological roles of neuroserpin has increased over the last years. In this review, we present an updated and critical revision of the current literature dealing with neuroserpin, covering all aspects of research including the expression and physiological roles of neuroserpin, both inside and outside the nervous system; its inhibitory and non-inhibitory mechanisms of action; the molecular structure of the monomeric and polymeric conformations of neuroserpin, including a detailed description of the polymerisation mechanism; and the involvement of neuroserpin in human disease, with particular emphasis on FENIB. Finally, we briefly discuss the identification by genome-wide screening of novel neuroserpin variants and their possible pathogenicity.

## Introduction

**Fig. 1:**
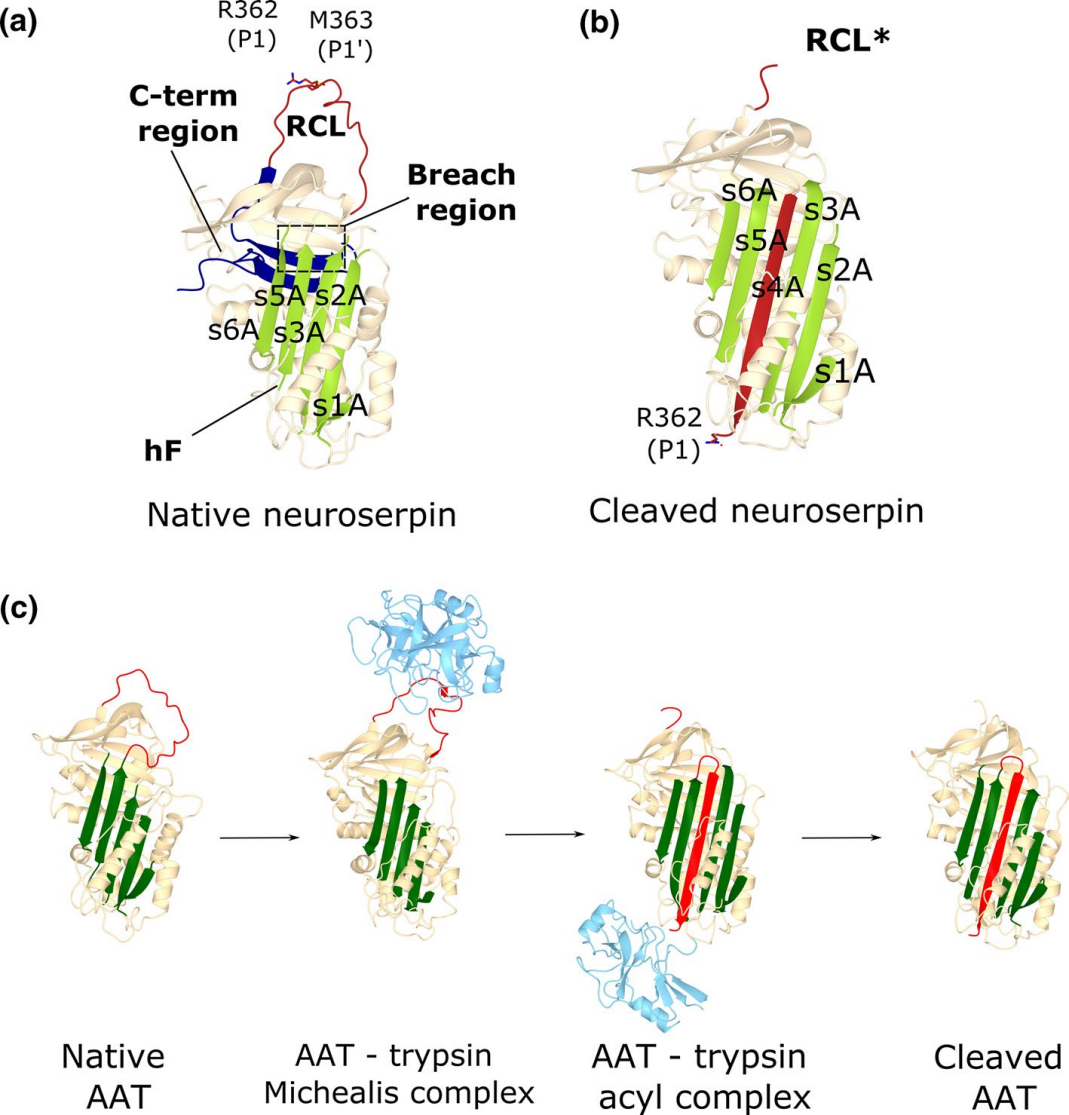
Neuroserpin structures and mechanism of inhibition. **a** The structure of native human neuroserpin (PDB 3F5N) shows the typical serpin fold: β-sheet A is shown in green, the RCL in red and the P1 and P1′ residues. The C-terminal region that gets swapped in polymers is shown in blue and the breach region, involved in β-sheet A opening, is highlighted by the dashed rectangle. **b** In cleaved human neuroserpin (PDB 3F02) the RCL loop (red) is proteolysed and inserted into β-sheet A (green) as an additional strand, s4A. **c** Native AAT (PDB 1QLP) presents the typical serpin fold with β-sheet A (green) and the exposed RCL loop (red). During inhibition, the target protease (trypsin, cyan) binds the RCL forming a Michaelis complex (PDB 1K9O) and cleaves the RCL at the P1–P1′ position. The RCL becomes inserted into β-sheet A and trypsin is translocated to the opposite side of the complex (PDB 1EZX). When the acyl-complex dissociates, cleaved AAT (PDB 2ACH) and trypsin are released

The superfamily of serpins (serine protease inhibitors) includes multiple proteins that exert varied functions [1]. Many of them are extracellular inhibitors of serine proteases and share a high degree of structural homology and a common mechanism of action. Indeed, these molecules are a beautiful example of the relationship between structure and function, and how this can be perverted to cause disease. Serpins fold to a metastable conformation to be able to change shape during protease inhibition [2]. The native conformation of neuroserpin shows the canonical features of inhibitory serpins (Fig. 1a): a five-stranded β-sheet A and an exposed, flexible reactive centre loop (RCL) that includes the scissile peptide bond (P1–P1’, so called for being the residues flanking the point of cleavage). These structural elements of serpins are essential for inhibiting their target proteases, but the structural flexibility needed to perform this function can be easily altered by point mutations to cause misfolding, leading to intermolecular linkage and formation of polymeric chains within the endoplasmic reticulum (ER) of cells [3]. Such polymers are not easy for the cells to deal with, so they accumulate and give rise to inclusion bodies that are the hallmark of diverse pathologies caused by this molecular mechanism: the serpinopathies [4]. Depending on the specific serpin, polymer deposition causes different clinical manifestations, which are due to both gain-of-toxic-function and loss-of-function events. Neuroserpin (SERPINI1) is an inhibitory serpin mainly expressed in the central nervous system (CNS), where it is involved in physiological processes including axonogenesis and synaptogenesis during embryonic development as well as synaptic plasticity and control of emotional behaviour in the adult [5]. It is also produced in other tissues across the body and new functions are being uncovered. We address here all aspects of neuroserpin biology, from its structure and inhibitory activity to its roles in physiology and disease, including a rare form of neurodegeneration that arises as a direct consequence of neuroserpin polymerisation.

## Identification and tissue expression of neuroserpin

Neuroserpin was first identified as a protein secreted from axons of cultured dorsal-root-ganglia neurons from chicken embryos [6]. The protein was classified based on the deduced amino acid sequence as a member of the serpin family of serine protease inhibitors [7]. Human neuroserpin is encoded by the *SERPINI1* gene (serpin_family_I_member_1; GeneID: 5274; OMIM 602,445), named according to the current classification of the serpin gene superfamily into phylogenetic clusters [8]. It is located on the human chromosome 3 at 3q26.1 and comprises nine exons, the first one being non-coding [9]. The open reading frame (reference mRNA sequence NM_005025.4) encodes for a 410 amino acid neuroserpin protein (UniProt: Q99574) that, after cleavage of the 18–19 amino acid signal peptide [10], has a molecular mass of 45 kDa, which increases after the addition of N-linked glycosylation as discussed later.

In the years following the identification of neuroserpin, its expression pattern was investigated in different organisms, from *Xenopus laevis* to mouse and human. Neuroserpin was mainly observed within the brain and spinal cord [11–14], and to a lesser extent in liver, kidney, pancreas and testis [12] as well as in cells of the immune system like macrophages, dendritic cells, T and B lymphocytes and natural killer cells [15, 16]. In the brain neuroserpin was first detected during the late stages of embryonic development, when newly generated neurons become postmitotic, start to migrate and differentiate. At this early stage, neuroserpin was detected homogeneously and at low levels in all brain regions. Neuroserpin levels increase perinatally and are maintained during adulthood, when the distribution of this serpin is enriched in the neocortex, hippocampus, amygdala and olfactory bulb, regions in which synaptic remodelling is associated with learning and memory [11]. At the cellular level, a similar trend has been observed: in the hippocampus, during development, the expression of neuroserpin is weak and the protein is ubiquitously found in many neurons of the pyramidal cell layer of the CA1 region and of the granule cell layer of the dentate gyrus, whereas in adulthood neuroserpin is selectively present, at higher levels, in a lower number of neurons [17]. These findings raised the possibility that neuroserpin could play a general role in neuronal maturation during development, while in the mature brain its function could be more restricted to specific processes. However, the analyses of neuroserpin expression conducted so far, which have excluded its synthesis by non-neuronal cells of the nervous system [11, 12, 17], have not detected the presence of neuroserpin in a particular neuronal subtype. Instead, it was found in several classes of glutamatergic pyramidal neurons as well as GABA-ergic interneurons, suggesting a pan-neuronal expression that disagrees with the assignment of a role in a specific type of neuron [11, 13, 18]. The physiological and pathological functions of neuroserpin have been investigated in detail in multiple model organisms, summarised in Table 1, and are discussed in dedicated sections below.

**Table 1:** Transgenic animal models for neuroserpin

Model organism	Genetic modification	Phenotype	References
*C. elegans*	Overexpression of wild type and H302R SRP-2	Formation of high molecular weight protein aggregates in H302R SRP-2 worms, phenotype aggravated by genetic deletion of HSF-1 and UPR pathways	Schipanski et al. [69]
*D. melanogaster*	Overexpression of chicken NS	Developmental defects (larval molting defects) caused by failure to progress through ecdysis resulting in significant increase in both larval and pupal lethality	Osterwalder et al. [157]
	Overexpression of wild type and P1–P1' (Pro–Pro) mutated wild type human NS	Ubiquitous expression of wild type NS was lethal for developing embryos, expression in the retina caused a rough eye phenotype. Phenotype rescued by co-expression of Abeta 1–42 peptide. Non-inhibitory P1–P1’-mutant was viable	Kinghorn et al. [60]
	Overexpression of mutated P1–P1' (Pro–Pro) version of wild type, S49P, S52R, H338R and G392E human NS	Intracellular accumulation of mutant NS in the brain of transgenic flies. Locomotor dysfunction in flies expressing mutant NS	Miranda et al. [66]
*D. rerio*	NS deficiency (knock-out)	Anxiety-like behaviour and deficits in axogenesis in the absence of locomotor defects	Han et al. [95]
	NS deficiency (knock-out)	Following hypoxic injury, developmental defects, reduced locomotion, neuronal loss, vascular malformation and oxidative stress more severe than in wild type animals	Han et al. [95]
*M. musculus*	Overexpression of wild type chicken NS	Reduced tPA activity in the brain. Following induction of focal ischemic stroke, smaller infarcts and attenuated microglial activation compared to wild type animals	Cinelli et al. [72]
	Overexpression of wild type chicken NS	Behavioural abnormalities (reduced centre exploration in the open-field test and neophobic response to novel objects)	Madani et al. [84]
	Overexpression of wild type human NS crossed with *pmn* mice (progressive motor neuronopathy)	Compared to *pmn* mice, decreased plasminogen activator activity in sciatic nerves and spinal cord, increased lifespan, stabilisation of motor behaviour, increased number of myelinated axons and rescued motoneuron number and size upon NS overexpression	Simonin et al. [158]
	Overexpression of wild type, S49P and S52R human NS	Intraneuronal NS-positive inclusion bodies accumulating in the ER in a mutation, age and dose-dependent manner in S49P and S52R mice, with clinical symptoms of the disease	Galliciotti et al. [108]
	Overexpression of wild type and G392E human NS	Age and dose-dependent accumulation of G392E NS in ER and lysosomes. Mutant mice were more susceptible to kainite-induced seizures	Takasawa et al. [113]
	Overexpression of wild type and S49P human NS	Correlation between mutant NS accumulation and neurodegeneration. Transient induction of the UPR in young mice	Schipanski et al. [69]
	Overexpression of wild type and S49P human NS	Transient inflammatory responses and UPR activation at middle stage of the disease, sequestration of UPR activators GRP78 and GRP94 in NS-positive inclusions	Lopez-Gonzalez et al. [118]
	Overexpression of wild type and S49P human NS	Increased expression of the postsynaptic protein PSD-95 in the hippocampus of S49P NS mice	Ingwersen et al. [159]
	NS deficiency (knock-out)	Unaltered tPA activity and behavioural abnormalities: reduced locomotor activity in novel environments, anxiety-like response on the O-maze, neophobic phenotype in the novel object test	Madani et al. [84]
	NS deficiency (knock-out) crossed with human APP-J20 transgenic mice	Rapid clearance of Abeta 1–42 injected into the frontal cortex in the absence of NS. Following crossing with human APP-J20 transgenic mice, decrease in amyloid-beta peptides, reduction in number and size of plaques, increased activity of tPA associated with plaques, rescue of spatial memory defects compared to J20 mice	Fabbro et al. [135]
	NS deficiency (knock-out)	Following induction of focal ischemic stroke, aggravated infarct size and neurological outcome and increased activation of proinflammatory microglia	Gelderblom et al., 2013 [123]
	NS deficiency (knock-out) and NS/tPA double deficient mice	Following kainic acid injection into the amygdala, reduced latency to seizure onset and generalisation, shorter mean time of survival and increase in blood–brain barrier permeability compared to wild type mice. NS/tPA double deficiency led to delayed latency to seizure onset and generalisation and protection from seizure-induced death	Fredriksson et al. [80]
	NS deficiency (knock-out)	In the hippocampus, decreased spine-synapse density, increased expression of the postsynaptic protein PSD-95, decreased synaptic potentiation and behavioural alterations in water maze test, contextual fear conditioning test and in social behaviour	Reumann et al. [97]
	NS deficiency (knock-out)	Deficits in developmental neurogenesis in the hippocampus (reduced proliferation of neuronal precursor cells and premature neuronal differentiation). Altered morphology of dendritic spines, increased expression and decreased proteolytic processing of the chondroitin sulphate proteoglycan aggrecan	Hermann et al. [17]
	NS deficiency (knock-out)	Unaltered neocortical lamination. Proteolytic processing of Reelin, expression of PAI-1, perineuronal net composition and synaptic proteome are unchanged in the neocortex	Kement et al. [18]

The table lists all the studies performed in animal models with modified neuroserpin (NS) expression found in the literature, indicating the species, the genetic modifications performed, the observed phenotypes and the corresponding references

## Structure, function and conformational flexibility of neuroserpin

Human neuroserpin shares a high sequence and structural homology with other members of the serpin superfamily, as well as the family’s canonical mechanism for protease inhibition, which renders serpins particularly flexible in terms of their tertiary structure. As a consequence of this, single serpin molecules (monomers) can adopt three different conformations: native, latent and cleaved.

### Native neuroserpin

Early biochemical work analysed the secondary structure of native neuroserpin by circular dichroism (CD) spectroscopy applied to wild type human neuroserpin [19–21] and its pathological variants Ser49Pro [19, 21] and Ser52Arg [20]. Several studies reported the structures of the native and cleaved conformations [22–24] (Fig. 1a, b) and confirmed that neuroserpin adopts the serpin fold, showing a core of three large β-sheets, including a five-stranded β-sheet A, nine α-helices and a long, mobile and well-exposed RCL. However, compared to other serpins, human neuroserpin presents some peculiarities. The omega-loop that connects strands 1 and 2 in β-sheet B, conserved in the superfamily, contains several glycine residues (Gly231, Gly236, and Gly237) that impose a particular flexible geometry, crucial for substrate binding. The region including helix F and strand 1 of β-sheet A also presents some aminoacidic substitutions when compared to the serpin consensus sequence: Asn162Gly, Leu162Lys and Val63Ile, which decrease the stability of helix F increasing the tendency to adopt the latent conformation, as also observed for plasminogen activator inhibitor 1 (PAI-1) and tengpin [25, 26]. Alterations of helix F dynamics can also reduce inhibitory activity and increase polymerisation [27]. Finally, the packing of β-sheet A is particularly tight thanks to an extra interaction between His338 and Ser340, which in other serpins is usually an alanine. Dynamic regions of neuroserpin such as the RCL and the omega-loop have been studied in detail by molecular dynamics simulations, hydrogen/deuterium exchange and optical spectroscopy. A molecular dynamics study [28] has evidenced a correlated collective movement of the protein related to the opening of the breach region (Fig. 1a) thought to be critical for neuroserpin inactivation [29, 30], as well as the formation of a persistent salt bridge between Glu289 on strand s2C and Arg362 on the RCL analogous to the archetypical serpin α_1_-antitrypsin (SERPINA1) [31].

### Molecular mechanism of inhibitory activity

Neuroserpin shares the inhibitory mechanism with the other members of the serpin superfamily, studied in detail for AAT (Fig. 1c). The target protease is recognised and loaded through the RCL, and a covalent complex between the active site of the protease and the P1 residue of the RCL is formed [32]. The protease cleaves the RCL through a canonical nucleophilic attack on the carbonyl C of the scissile peptide bond between P1 and P1’ (Fig. 1a, c), forming an acyl-complex. The cleavage causes a major structural rearrangement of the serpin: the RCL is inserted as β-strand 4 into β-sheet A, and the protease is translocated to the opposite side of the serpin-protease complex (Fig. 1c) [33]. Typically, such conformational change causes the disruption of the active site in the serine protease and prevents the hydrolysis of the acyl-bond, rendering the covalent complex extremely stable over time [34]. The complex is degraded intracellularly after its cellular internalization, usually mediated by interaction with the low-density lipoprotein receptor-related protein (LRP) [35, 36].

This inhibitory mechanism is conserved for neuroserpin and tPA, which hydrolyses neuroserpin’s RCL at Arg362, but the acyl-complex is peculiarly short-lived [19, 23, 37]. As a consequence, the inhibition of tPA is only transient and in vitro, when the complex dissociates, active tPA and cleaved, inactive neuroserpin molecules are released. Despite this, some observations suggest that this interaction may be more stable in vivo. An electrophoretic band compatible with a neuroserpin-tPA complex was identified in murine brain homogenate [12], and the internalization of both neuroserpin and the neuroserpin-tPA complex has been reported in murine primary cortical cells through interaction with LRP [38]. Compared to other serpins, strands sC1 and sC2, the loop between helices C and D, and helix E of neuroserpin contribute to tPA recruitment and to stabilization of the neuroserpin-tPA complex, suggesting that the primary sequence has a role in regulating the stability of the acyl-complex [39]. Altering the RCL conformation by disruption of the salt bridge between residues R362 and E289 contributes to the fragility of the neuroserpin-tPA complex [30]. A detailed study has also shown that pH has an important role in substrate recognition and deacylation rates during neuroserpin’s inhibition of tPA, which is different between the single-chain and two-chain forms of tPA [40]. It should be noted that many of these studies have been performed using recombinant neuroserpin lacking *N*-glycans, but a recent report shows that N-glycosylation slightly improves the inhibitory activity of neuroserpin against tPA [41].

### Cleaved neuroserpin

The serpin fold is generally maintained in the cleaved conformation of neuroserpin, as observed in the crystal structure (Fig. 1b) [22, 23], with the cleaved RCL nested as strand 4 in β-sheet A to form a sixth-stranded β-sheet. This transition of the RCL, from solvent-exposed to buried, occurs physiologically and is pivotal for the exertion of the inhibitory activity of neuroserpin. The RCL segment after the cleavage site remains exposed, as in native neuroserpin. In the crystal structure, the C-terminal stretch of the RCL is visible, being stabilised by contacts to the distal part of β-sheet A [23].

### Latent neuroserpin

A crystal structure of latent neuroserpin has not been reported, but it is likely similar to that of PAI-1 [42], with RCL insertion into β-sheet A in the absence of cleavage. A model has been proposed based on the structures of latent α_1_-antitrypsin and cleaved neuroserpin, and findings from in vitro spectroscopic fingerprinting [28] are in agreement with the formation of the typical six-stranded β-sheet A. The transition to the latent conformation is reported to be an auto-regulatory mechanism [24], while in vitro it can be induced by heating. Wild type neuroserpin has been found to undergo a conformational transition at 56 °C, with concomitant formation of the latent and polymeric conformers, and a second transition at 85 °C, indicative of the formation of hyper-stable monomeric and polymeric conformations, further increasing the complexity of neuroserpin’s conformational landscape [43].

## Polymerisation of neuroserpin

The metastable nature of their native conformation renders serpins prone to establish intermolecular interactions that result in non-covalent but highly stable links between two or more serpin molecules to form polymers [44]. These can be made in *vitro* by applying heat or chemical denaturants to wild type or mutant variants of neuroserpin [45], and are formed in vivo as a consequence of point mutations that destabilise neuroserpin, leading to a rare neurodegenerative dementia called FENIB (familial encephalopathy with neuroserpin inclusion bodies, discussed in detail below). Two FENIB-causing variants, Ser49Pro and Ser52Arg neuroserpin, have been expressed in vitro and purified, but due to their instability in solution only a partial biochemical characterisation has been achieved. Both variants showed a strongly reduced ability to inhibit tPA, together with a high tendency to form polymers [19, 21]. Urea gradient electrophoresis and emission spectroscopy showed transition points at 6.4, 6.3 and 5.3 M urea for wild type, Ser52Arg and Ser49Pro neuroserpin, respectively, revealing Ser52Arg to be more stable than Ser49Pro neuroserpin even though the FENIB phenotype associated to the Ser52Arg mutation is more severe [20]. An additional unfolding step was evident at nearly 1 M urea, suggestive of a polymer-competent intermediate conformation [20, 28, 43].

### Polymer structure

The molecular details of neuroserpin polymers are not known at high resolution, adding to the debate in the serpin field about the nature and structure of serpin polymers. Alpha-1-antitrypsin polymers, usually taken as reference for other serpins, were initially thought to be formed by the insertion of the RCL of one molecule into β-sheet A of another (‘loop-sheet’ model) [46], while later work based on crystallisation approaches has proposed two novel mechanisms based on different domain swaps [47, 48]. Of these, the most recent one (‘C-terminal’ model) asserts that an intermolecular link is formed by insertion of the non-properly folded C-terminus (Fig. 1a) of a donor protein into an acceptor protein. This model is currently accepted for Z α_1_-antitrypsin polymers, also based on recent findings obtained by electron microscopy imaging of polymers extracted from liver [49]. This model provides a straightforward explanation for the remarkable flexibility of α_1_-antitrypsin polymers recently observed [50]. While an analogous flexibility has been suggested for neuroserpin polymers [51], there is no high-resolution structure currently available for them.

The first image of neuroserpin polymers was obtained by electron microscopy of protein aggregates released after sonication of intraneuronal inclusion bodies from a patient’s brain. They showed as entangled fibrils and short-chain filaments formed by Ser49Pro neuroserpin [52]. More recently, neuroserpin polymers made in vitro by heating were imaged by electron microscopy [43] and by atomic force microscopy [53], closely resembling the ones extracted from patients. Most of the molecular research about neuroserpin polymers is based on samples prepared in vitro, relying on the observations that neuroserpin polymers extracted from cultured cells and those formed by heating exhibited identical polymer ladders after non-denaturing electrophoresis and similar reactivities to monoclonal antibodies [20, 54]. Experiments using purified wild type, Ser49Pro and Ser52Arg neuroserpin revealed a faster polymerisation for the Ser52Arg mutant [20] and the ability of latent Ser49Pro neuroserpin to form polymers [21], a behaviour never observed for wild type or other variants of neuroserpin, suggesting a partially open breach region and partial RCL insertion even in the native conformation for this variant. In contrast, CD and photoluminescence experiments on neuroserpin refolding have shown that folding intermediates are more polymerogenic than the folded conformations [55], supporting the formation of polymers by domain swapping during protein folding within the ER. The panorama has been further enriched by the discovery of another type of hyper-stable neuroserpin polymeric species formed by heating at a higher temperature [43]. Neuroserpin polymerisation at both intermediate (45 °C) and high temperature (85 °C) occurred as a two-state transition from the native to the polymer state with different extents of multiple-stranded β-sheet elements [56], compatible with partial or complete opening of the breach region [21].

The importance of specific protein regions in neuroserpin polymer formation has been studied by introducing selected point mutations. Helix F and β-sheet A were found to be crucial, since restoring the aminoacidic composition to the serpin consensus sequence increased the stability and reduced the polymerization tendency of neuroserpin [24]. In agreement with this, disrupting the interaction between helix F and β-sheet A led to a higher polymerisation rate or a tendency to precipitate [27]. Finally, mutations in helix B and β-sheet B reduced the propensity to polymerization but promoted the transition to the latent conformation [57]. Regarding the effects of environmental conditions on polymer formation, inhibition by low pH has been observed and explained by the protonation of His residues 119 and 138 that normally form stabilising hydrogen bonds while, in contrast, the protonation of His338 disrupts a hydrogen bond reducing conformational stability [58]. The resistance of neuroserpin against polymer formation in acidic conditions, opposed to the behaviour of other serpins, may have evolved to avoid polymerisation within the regulated secretory vesicles into which neuroserpin is accumulated before secretion. Finally, some sugar and alcohol molecules [59] and the β-amyloid peptides Aβ-40 and Aβ-42 [60, 61] are able to reduce polymer formation, probably through direct interaction with neuroserpin.

### Polymerisation mechanisms

**Fig. 2:**
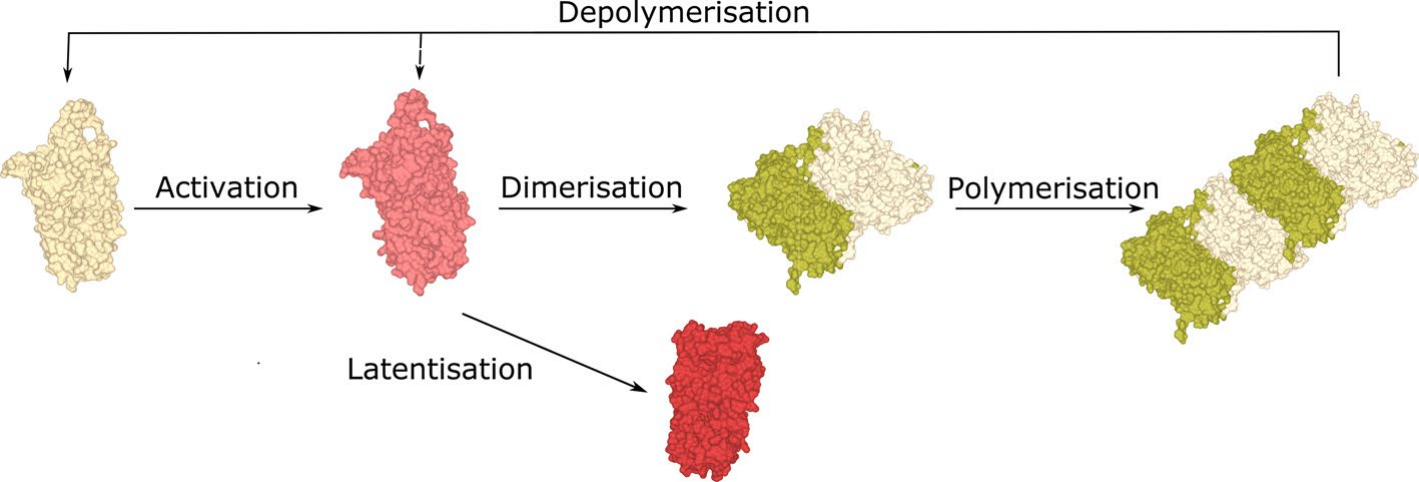
Mechanism of neuroserpin polymerisation. A native neuroserpin monomer can convert to an activated intermediate conformation that is able to reach the inactive latent form or to associate with another monomer to form a dimer, and initiate polymerisation. Eventually, polymers may undergo fragmentation

Our current understanding about neuroserpin polymer formation is based on two in vitro studies. The first one used a single-molecule approach, two colours coincidence detection (TCCD), together with non-denaturing electrophoresis to address the first stages of polymerisation [61]. The second one employed several techniques including light scattering, photoluminescence and time-lapsed size exclusion chromatography [51]. From these studies, the following processes have been deduced for neuroserpin polymerisation (Fig. 2): (i) activation: polymer formation requires the initial activation of an intermediate polymerogenic monomer (activation rate in the order of 10^–5^ s^−1^); further kinetic experiments confirmed that the overall polymerisation rate is in the same order as the activation rate, which is, therefore, the rate-limiting step [56]; (ii) latentisation: the activated monomer may change into a stable latent conformation (rate in the order of 10^–2^ s^−1^); (iii) dimerisation: the activated monomer may associate either with another activated monomer or with a native monomer to form a dimer, with a rate in the order of 10^3^ M^−1^ s^−1^ [61] or one order of magnitude lower [51]; (iv) monomer addition: each polymer chain may elongate by single monomer addition (estimated rate of 10^1^ M^−1^ s^−1^); (v) polymer association: already formed polymers may associate with other polymers, only at polymer ends, without other secondary processes such as lateral association or branching [51]; the kinetic rate in the late stages of polymerisation was still of the same order as the monomer addition rate; (vi) fragmentation: although serpin polymers are considerably stable, light scattering kinetics analysis revealed a reduction of the average aggregate mass [51], providing the first observation of an explicit fragmentation process during protein aggregation. Interestingly, while fragmentation typically enhances protein aggregation [62], in this case it had the opposite effect: tempering polymer formation, likely due to the simultaneous occurrence of latentisation. The latter process seems particularly important to introduce novel therapeutic strategies based not only upon the inhibition of polymer formation, but also on the disruption of already formed polymers and on the clearance of excess neuroserpin, both in the monomeric and polymeric forms. Indeed, a theoretical study supports this perspective, showing that neuroserpin aggregation within the ER may be modulated by the ratio between the production and clearance rates, and that polymerisation may be triggered by a non-equilibrium phase transition [63]. Further experimental support in this direction comes from the observation that polymerisation is enhanced by reducing the cellular levels of cholesterol, and hence the ability to form vesicles needed for neuroserpin clearance [64].

Recent studies have investigated the ability of embelin, a naturally occurring para-benzoquinone isolated from dried berries of *Embelia ribes*, to interfere with neuroserpin polymerisation. Embelin was shown to bind all conformers of neuroserpin and to be able to prevent polymer formation in vitro, promoting the formation of small, soluble oligomers; moreover, embelin addition to preformed polymers caused their disassemble to soluble oligomers (Fig. 2B, oligomers_NS-EMB_) [53]. Unfortunately, several attempts to ameliorate the solubility of embelin and its affinity for neuroserpin have shown that even minor chemical modifications result in a marked reduction of its antipolymerisation activity [65].

## The importance of being earnestly glycosylated

In addition to point mutations, both the pathological ones and the ones specifically designed to probe the stability of neuroserpin, another endogenous factor is extremely important in determining molecular stability and hence in preventing polymerisation: glycosylation. Sequence analysis of human neuroserpin revealed the presence of three potential N-glycosylation sites: Asn157, Asn321 and Asn401 [9] (Fig. 4a, left panel), and all three appeared to be glycosylated in Ser52Arg neuroserpin extracted from Collins’ bodies of a FENIB patient [10]. The presence of *N*-glycans has also been demonstrated in several cellular expression systems [54, 66–68], but its impact on the stability of neuroserpin has been evaluated only recently. The first observation described the importance of *N*-glycans for the quality control of neuroserpin, in particular for directing the protein to ER associated degradation (ERAD) through a direct interaction with the lectin OS-9 [69]. A study based on directed mutagenesis revealed that wild type neuroserpin is glycosylated at the Asn157 and Asn321 sites, while the pathological variant Gly392Glu is also partially glycosylated at residue Asn401 [70]. Moreover, the same study showed that perturbation of the glycosylation profile leads to increased polymerisation of wild type neuroserpin, likely due to the loss of steric exclusion and conformational fluctuations. Most of the in vitro studies about conformational stability and polymerisation of neuroserpin have been performed using bacterial recombinant protein, thus overlooking the effects of glycosylation. Recently, a new expression model based on a modified *Leishmania* strain has been set up to produce neuroserpin with mammalian-like glycosylation [41]. This in vitro work has confirmed a decreased tendency to polymerisation, a higher propensity to acquire the latent conformation, and a slight increase in inhibitory activity for the glycosylated protein.

## Physiological roles of neuroserpin

### Physiological roles of neuroserpin in the nervous system

The search for neuroserpin’s function started right after its identification. Analysis of its RCL revealed a higher degree of similarity to inhibitory rather than non-inhibitory serpins [7]. With an arginine and a methionine, respectively, at positions P1 and P1’ of the RCL (Fig. 1a), it was hypothesized that neuroserpin would target trypsin-like serine proteases. This was indeed demonstrated in different in vitro studies showing complex formation and inhibition of the proteolytic activity of tissue-type plasminogen activator (tPA), and to a lesser extent of urokinase plasminogen activator (uPA), trypsin and plasmin [11, 12, 71]. Complex formation with tPA, but not with uPA, has been later demonstrated in vivo in the brain of rodents overexpressing neuroserpin [72, 73], and recent work has shown that neuroserpin can discriminate between one- and two-chain tPA [40]. The serine protease tPA has a prominent role in the fibrinolytic cascade, where it activates plasminogen to plasmin, but it is also highly expressed in the CNS, with a pattern overlapping that of neuroserpin [18, 74]. tPA participates in different physiological processes, such as brain development, neuronal outgrowth and synaptic plasticity [75–77]. Furthermore, tPA plays a role in pathological conditions of the CNS by contributing to excitotoxic neuronal degeneration and neuronal damage following cerebral ischemia, through the regulation of the permeability of the neurovascular compartment [78–80]. The activity of tPA is reduced in the brains of mice overexpressing neuroserpin [72]. Moreover, administration of neuroserpin blocked tPA-dependent visual cortical plasticity in adult mice, and tPA-dependent cell proliferation, interaction and migration in vitro [81–83]. However, zymographic analysis of neuroserpin-deficient brain tissue showed unaltered tPA activity [84], and compensation by another serpin has often been hypothesized. Neuroserpin protects neurons against tPA-mediated injury during pathologic events involving cerebral ischemia [85–87], but this neuroprotective role was found to be at least partially independent from tPA inhibition and rather mediated by inhibition of plasmin-induced excitotoxin cell death [88, 89], suggesting that alternative targets may exist. Furthermore, the unstable, short-lived nature of the complex formed in vitro between neuroserpin and tPA [38], together with the fact that a mutant form of neuroserpin lacking inhibitory activity showed the same ability as the wild type protein in regulating N-cadherin-dependent cell–cell adhesion [90], point to a possible function for neuroserpin beyond its anti-protease role. Alternative, non-inhibitory mechanisms for neuroserpin are often discussed in the field, for instance those mediated through interaction with an extracellular receptor. Indeed, neuroserpin has been shown to bind to and be internalised by LRP [37], a protein known to play a role in neurodevelopment and synaptic function [91, 92].

**Fig. 3:**
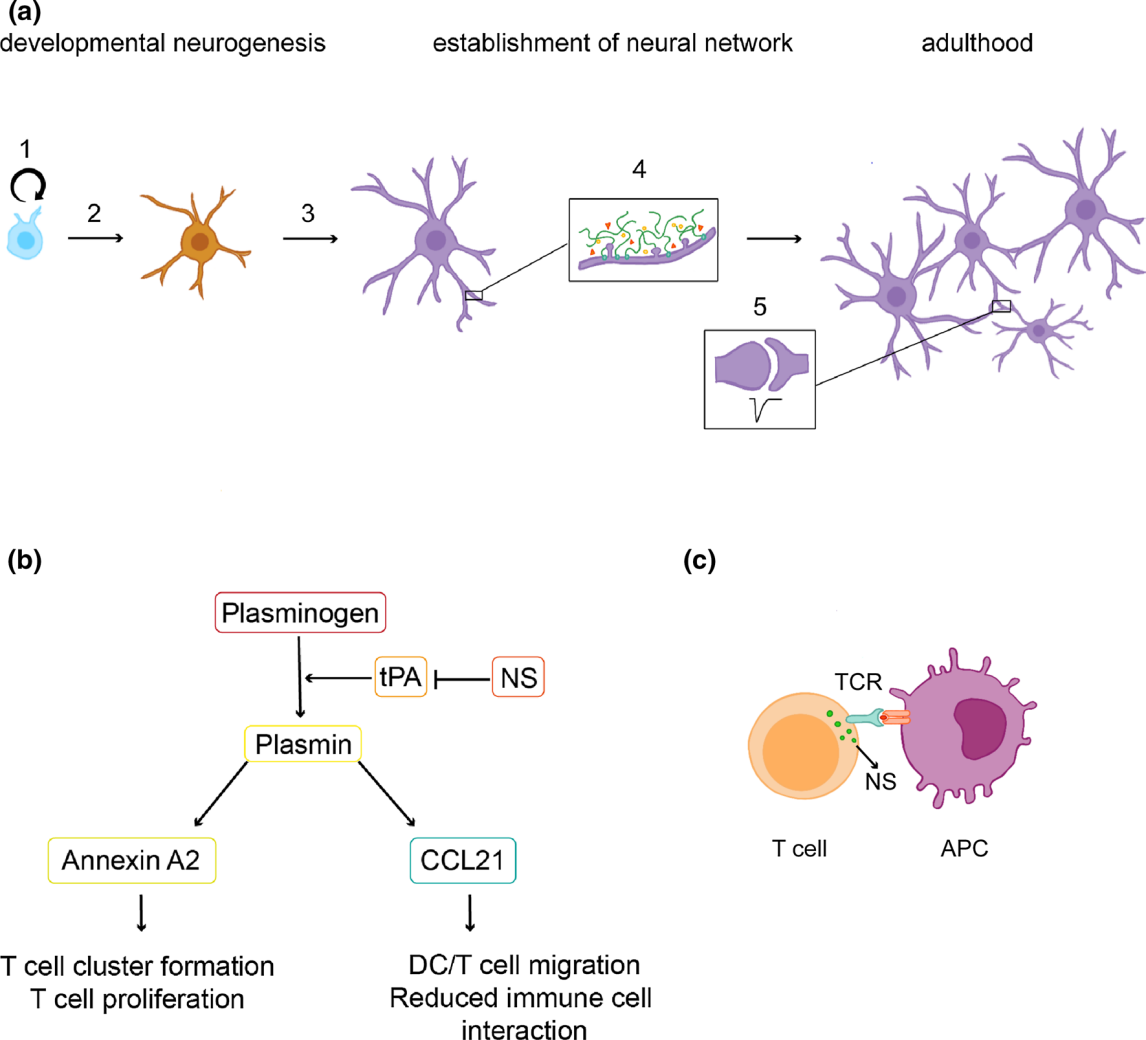
Physiological roles of neuroserpin. **a** During brain development, neuroserpin plays a role in hippocampal neurogenesis by modulating neuronal precursor proliferation (1) and differentiation (2). Moreover, whereas in vitro studies point to a role in dendritic arborisation (3), in the murine hippocampus neuroserpin regulates maturation of dendritic spines and their surrounding perineuronal net (4). In the adult mouse brain, deficits in synaptic plasticity have been observed (5), correlating with behavioural abnormalities in hippocampal-dependent tasks. **b** Annexin A2 and CCL21 are both plasmin substrates whose cleavage regulates several processes in the immune system. Neuroserpin is thought to play a role in these pathways by modulating the tPA-dependent proteolytic activation of plasmin from plasminogen. **c** Upon T cell activation by antigen presentation, neuroserpin-positive vesicles are translocated to the immunological synapse and neuroserpin is rapidly released; *TCR* T cell receptor; *APC* antigen presenting cell

Although in some cases the mechanisms by which neuroserpin exerts its roles remain to be elucidated, analysis of cultured cells and animal model brains with over- or under-expression or deficient in this serpin have provided important information about its function (Fig. 3a and Table 1). A high perinatal expression throughout the developing nervous system supports a role for neuroserpin in late developmental processes involving neuronal differentiation, synaptogenesis and refinement of synaptic connections. The absence of neuroserpin during brain formation in knock-out mice resulted in deficient neurogenesis, with reduced proliferation of neuronal precursors in the granular cell layer of the dentate gyrus [17]. This was accompanied by early neuronal differentiation and resulted in decreased cellularity of the adult dentate gyrus, demonstrating a key role for neuroserpin in regulating the formation of the hippocampus. The organization of the neural network, encompassing neurite outgrowth and establishment of synaptic connections, requires the activity of neuroserpin as well. Experiments with neuroendocrine cells revealed that neuroserpin regulates neurite extension. Overexpression of neuroserpin induced the expression of neurite-like processes in AtT-20 pituitary cells, in the absence of increased levels of the neuroserpin-tPA complex, suggesting a tPA-independent mechanism [93]. In pheochromocytoma (PC12) cells, neuroserpin was observed in dense-cored secretory vesicles located at the growth cones and its levels correlated to the total length of neurite outgrowth [94]. In vivo, ablation of the neuroserpin gene in zebrafish larvae resulted in defects in the extension of axons in primary motoneurons [95]. Studies in primary neurons showed that neuroserpin influences the formation of dendritic spines, small protrusions emerging from dendrites that represent the postsynaptic elements of excitatory synapses. In particular, overexpression of neuroserpin in primary rat neurons increased spine density and caused a reduction in spine head size [96]. These observations are in line with the alterations detected in the hippocampus of juvenile neuroserpin-deficient mice, where increased size of dendritic spine heads has been observed [17]. The same animals also displayed defects in the perineuronal net, a component of the brain extracellular matrix that enwraps certain neurons and critically controls synaptic maturation [17].

During adulthood, higher levels of neuroserpin expression in brain regions involved in synaptic plasticity prompted the investigation of a possible involvement of the serpin in this process (Fig. 3). For the hippocampus, this was addressed by analysing rodents either lacking or overexpressing neuroserpin. In neuroserpin-knock-out mice, alterations were observed at morphological, functional and behavioural levels, with decreased spine-synapse density resulting in reduced synaptic potentiation and deficits in hippocampal-dependent cognitive and social functions [84, 97]. Overexpression of neuroserpin in rodent brains caused different scenarios: whereas transgenic overexpression in neurons starting between postnatal days 4–10 translated in a neophobic phenotype [84], targeted injection of a viral vector expressing neuroserpin resulted in overexpression of the serpin in the rat dorsal hippocampus but failed to induce behavioural deficits [73]. This discrepancy may result from differences in spatial and temporal overexpression of neuroserpin, thus highlighting the importance of a regulated expression of this serpin.

### Physiological roles of neuroserpin outside the nervous system

Several studies have uncovered physiological functions for neuroserpin outside the nervous system, specifically in immune cell function. This serpin was found to be highly expressed and secreted by monocyte-derived macrophages and dendritic cells following differentiation [15], as well as by T and B lymphocytes and natural killer cells [16]. By regulating the cleavage of the chemokine CCL21 (C–C motif ligand 21), neuroserpin controlled the migratory capacity of T cells and dendritic cells [82], and by modulating the cleavage of annexin A2, neuroserpin altered T cell-T cell interaction, proliferation and cluster formation [83]. Interestingly, both proteins are cleaved by plasmin, and the inhibitory activity of neuroserpin (presumably against tPA) is required to modulate both events, since an RCL mutant was proven to be ineffective (Fig. 3b). Moreover, upon T cell activation, neuroserpin was intracellularly translocated to the immunological synapse and rapidly secreted from T cells [16] (Fig. 3c). This process was followed by downregulation of neuroserpin expression and concomitant upregulation of its inhibitory target tPA; thus, similarly to neuronal synapses, a role for neuroserpin in the regulation of extracellular proteolysis can be hypothesized at the immunological synapse as well. Future studies are needed to characterise the downstream targets of these proteolytic processes. A few candidates have been suggested [16], including FasL and L1CAM that modulate metastatic colonisation of the brain [98], and MMP9 that regulates extracellular matrix degradation as well as the levels of molecules involved in inflammatory mechanisms [99].

Finally, a role for neuroserpin has also been described in vascular inflammation and atherosclerosis [100]. In a mouse aortic allograft transplant model, administration of neuroserpin immediately after surgery exerted an anti-inflammatory activity by reducing plaque growth, CD3^+^ T cell invasion and T-helper cell activation four weeks after transplantation, opening a new avenue of research for neuroserpin function and therapeutical use.

## Roles of neuroserpin in human disease

### Familial encephalopathy with neuroserpin inclusion bodies (FENIB)

**Fig. 4:**
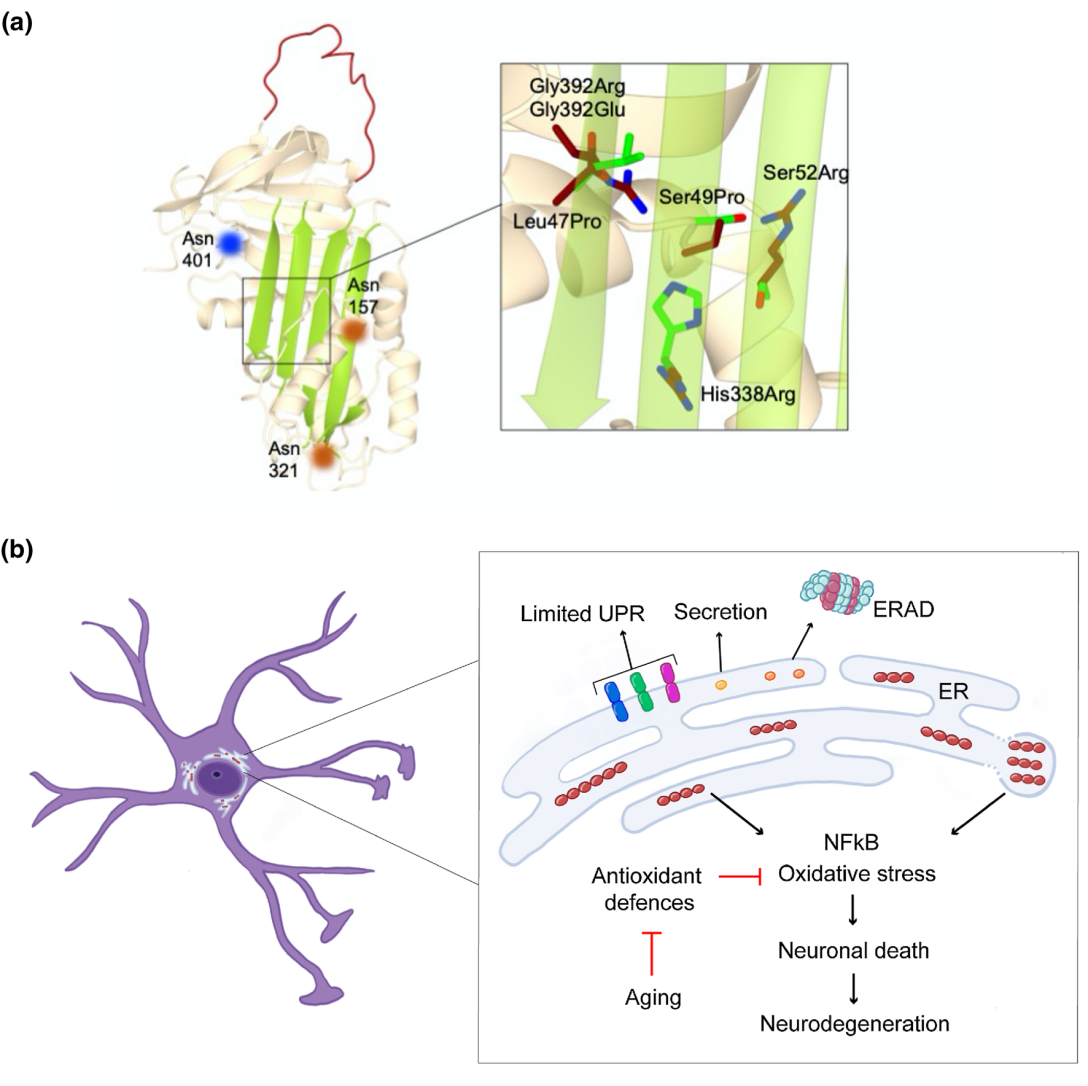
N-glycosylation of neuroserpin, FENIB related mutations and toxicity mechanisms of polymerogenic neuroserpin. **a** The positions of the two physiological N-glycosylation sites Asn157 and Asn321 (orange spots) and the aberrant site Asn401 (blue spot) are shown on the structure of human native neuroserpin (left panel, PDB 3F5N). The box on the right focuses on β-sheet A to show the six pathological mutations known to cause FENIB, with the wild type and mutated residues colored in green and dark red, respectively. **b** Cellular responses to the presence of polymerogenic mutant neuroserpin. The monomeric forms are in part secreted, in part degraded by the proteasome through ERAD and in part incorporated into polymeric chains that can be found in tubular ER and ER-derived inclusions. The expression of polymerogenic neuroserpin causes NFκB activation and chronic oxidative stress, leading to neuronal death and neurodegeneration. This is probably more pronounced with aging, due to a weakening of the antioxidant defences

Point mutations in neuroserpin cause a rare autosomal dominant form of neurodegeneration called FENIB [52, 101], with clinical manifestations that include dementia, myoclonic seizures and epilepsy [102, 103]. A key pathological finding in FENIB is the diffused presence of eosinophilic, PAS (periodic acid-Schiff)-positive and diastase-resistant neuronal inclusions, named Collins bodies, distributed mainly throughout the grey matter of the cerebral cortex and the subcortical nuclei, especially the *substantia nigra* [101, 104], but also in the spinal cord and dorsal root ganglia [105]. The analysis of Collins bodies showed that they are composed of neuroserpin polymers with identical morphology to polymers obtained from hepatocytes of an α_1_-antitrypsin deficiency patient, establishing a common molecular mechanism for both pathologies [101]. Biochemical analysis of Ser52Arg neuroserpin polymers from a heterozygous FENIB brain also showed that the wild type protein was not retained in the inclusions [10], a result later supported by studies in cell culture [54]. This is in contrast with studies showing a mild degree of heteropolymer formation by wild type and mutant Z (Glu342Lys) α_1_-antitrypsin both in cell cultures [106] and in polymers extracted from the liver [107]. Clinical findings in human patients [104] and studies in transgenic animals [66, 108] and cell culture models of FENIB [54, 66] indicate that the degree of neuroserpin retention within the ER is directly proportional to the propensity to form polymers and the severity of the clinical phenotype caused by each mutation, and inversely correlated to the age of onset of clinical symptoms. All six point mutations identified so far (Fig. 4a, right panel), Ser49Pro (*Syracuse*,[52, 101]), Ser52Arg (*Portland*, [52, 101, 109]), His338Arg [104], Gly392Glu [104, 110], Gly392Arg [111] and Leu47Pro [105] present in heterozygosis and lead to polymerisation of neuroserpin, slowing the trafficking of the mutant protein from the ER and causing polymer accumulation in its lumen, as established by electron microscopic analyses of post-mortem brains [101, 109] and by studies in cell models of disease [54, 66, 68–70, 112]. ER retention of mutant neuroserpin causes neuronal damage through a toxic gain-of-function mechanism, as demonstrated in mouse and fly models of FENIB where overexpression of human polymerogenic neuroserpin causes neurological symptoms reminiscent of those seen in FENIB patients [66, 108, 113]. The concomitant reduction of neuroserpin secretion may be responsible for some of the clinical manifestations of FENIB, in particular epilepsy due to reduced inhibition of tPA [103]. The first cellular response to the presence of mutant neuroserpin (Fig. 4b) is to remove it by ERAD [54, 67, 114], through a mechanism that involves Hrd1 [115] and the lectin OS-9 [69] and connects with the pathway for cholesterol biosynthesis [112]. Although part of the aberrant protein is thus eliminated, an important fraction of the polymerogenic protein escapes degradation, forms polymers and accumulates within the ER, giving rise to the inclusion bodies typical of the serpinopathies. As seen before for polymers of α_1_-antitrypsin [116, 117], neuroserpin polymer accumulation does not generally trigger the unfolded protein response (UPR), the canonical signalling pathway that increases the cell’s capacity to deal with misfolded ER proteins [67, 112], although a few reports describe a limited activation in vivo [69, 118]. In contrast, α_1_-antitrypsin [116, 117] and neuroserpin [67] polymers activate an ER stress pathway based on calcium-dependent activation of NFκB (nuclear factor κ-light-chain-enhancer of activated B cells) that can promote inflammation and cell death, the ER overload response (EOR, [119]). These evidences highlight the peculiarities of FENIB when compared to other neurodegenerative diseases like Alzheimer’s and Huntington’s disease and amyotrophic lateral sclerosis, where protein misfolding is associated to activation of UPR and ERAD [112]. A recent study in neural progenitor stem cells has also reported the upregulation of antioxidant enzymes and increased sensitivity to oxidant insults in response to accumulation of polymerogenic neuroserpin, supporting a role for oxidative stress in FENIB [68], in agreement with previous studies on α_1_-antitrypsin deficiency in mice [120] and in patients [121].

### Role of neuroserpin in ischemic syndromes

One of the earliest connections of neuroserpin with human pathology was its marked association with ischemic syndromes, initially studied in animal models reporting that neuroserpin expression rapidly decreased within the ischemic core following stroke, while it increased in the region surrounding the lesion (penumbra) [85, 122]. Neuroserpin exerted a neuroprotective action, since its administration by intracerebral injection or by overexpression in a transgenic mouse model resulted in increased neuronal survival and reduction in stroke volume, blood–brain barrier (BBB) leakage and consequent brain oedema [72, 85, 122]. Conversely, middle cerebral artery occlusion (MCAO) in neuroserpin-deficient mice resulted in a more severe phenotype with increased infarct size and aggravated neurological outcome [123]. The mechanism underlying these effects is still a matter of debate. Early studies proved that neuroserpin reduced the transient, deleterious increase in tPA activity following ischemic insult [72, 85], but later work spoke in favour of a tPA-dependent but plasminogen-independent effect on vascular permeability and suggested an LRP-mediated cell signalling mechanism [124]. A few years later, a study with tPA-deficient mice demonstrated that neuroserpin inhibition of tPA was dispensable for its neuroprotective function, and a mechanism involving regulation of plasmin-mediated excitotoxin-induced cell death was proposed [88]. Several in vitro studies have also provided variegated results. Cell culture models subjected to oxygen and glucose deprivation support tPA-dependent neuroprotection, most likely by regulating the levels of molecules involved in the inflammatory response and in extracellular matrix degradation like MMP9 [99], as well as increased survival of neurons and astrocytes through modulation of inflammatory pathways [125, 126]. In contrast, neuroserpin preserves retinal function by attenuating neuronal loss after retinal ischemic/reperfusion-induced injury in both wild type and tPA-deficient mice, suggesting a tPA-independent neuroprotective mechanism [89]. Clinical data from ischemic stroke patients also support a neuroprotective role for neuroserpin: higher serum levels of this serpin were associated with a better outcome after stroke, while their decrease over time was associated to increased excitotoxicity, inflammation and BBB disruption [127, 128]. These data, together with the observation that neuroserpin treatment increased the therapeutical window for tPA administration after MCAO in mice [122, 129], support a therapeutic application for neuroserpin in stroke patients.

### Role of neuroserpin in neuropsychiatric disorders

Over the years, a role for neuroserpin has been described not only in FENIB, but also in other severe neurodegenerative and neuropsychiatric disorders. Altered neuroserpin expression in transgenic mice lacking or overexpressing this serpin has been shown to cause increased phobic and anxiety-like behaviours [84] and cognitive and sociability deficits in the absence of neuroserpin [97]. Similarly, neuroserpin deficiency in zebrafish larvae led to anxiety-like behaviour in the absence of locomotor defects [95]. In humans, upregulation [130, 131] or downregulation [132] of neuroserpin mRNA has been found in brain tissue and iPS-derived neurons of patients suffering from schizophrenia, although a recent study did not find these alterations in similar conditions [97]. Also, a significant reduction of neuroserpin mRNA levels was observed in a rat model of stress and depression and in peripheral blood mononuclear cells (PBMCs) from patients with first-episode depression [133], supporting the involvement of neuroserpin in these neuropsychiatric disorders.

### Role of neuroserpin in Alzheimer’s disease

Several studies support a role for neuroserpin in Alzheimer’s disease (AD) pathogenesis. It was first described that neuroserpin was associated to Aβ plaques in the brain of AD patients, and in vitro work showed that neuroserpin formed a 1:1 binary complex with the N-terminal or middle part of the Aβ(1–42) peptide, leading to the inactivation of neuroserpin as an inhibitor of tPA, preventing the heat-induced polymerization of neuroserpin, altering Aβ oligomerisation to a non-fibrillary species, and rendering Aβ peptides less toxic to neuronal cells in culture [60]. The presence of neuroserpin in Aβ plaques was later confirmed, and neuroserpin was shown to be upregulated in the brain of AD patients, leading to the hypothesis that increased inhibition of tPA and reduced plasmin activity decreased Aβ amyloid clearance and maybe caused synaptic alterations [134]. In a follow-up study, neuroserpin-deficient AD transgenic mice showed a strong reduction in Aβ peptide and plaques and a concomitant improvement in AD-related cognitive deficits such as spatial memory [135], supporting a role for the neuroserpin-tPA axis in Aβ amyloid deposition and the loss of cognitive abilities. It has also been shown that AD patients present higher levels of neuroserpin in the cerebrospinal fluid (CSF), in association with higher tau protein levels, suggesting its use as a potentially useful marker for clinical diagnosis of the pathology [136]. In contrast, a conflicting report found that neuroserpin was significantly reduced in the frontal and temporal cortex in AD compared to control brains [137]. A small but significant increase in neuroserpin levels has also been found in subjects with mild cognitive impairment [138], and in AD brains in correlation with elevated thyroid hormone receptor β1 expression, providing evidence for a potential relationship between thyroid problems, AD and neuroserpin [139]. Other studies have correlated the upregulation of neuroserpin that, together with the decreased expression of tPA, has been observed at advanced stages of amyloid pathology in AD patients and in a transgenic rat model of AD, with dysregulation of the nerve growth factor (NGF) and brain derived neurotrophic factor (BDNF) metabolic pathways [140, 141]. Altered NGF maturation, characterised by pro-NGF accumulation and reduced presence of mature NGF, together with increased NGF degradation, caused by enhanced activity of the matrix metalloprotease MMP9 in AD, have been suggested to impair the trophic support of basal forebrain cholinergic neurons, as demonstrated by the reduction of cortical and hippocampal cholinergic synapses in transgenic AD rats. This highlights the importance of the neuroserpin-tPA protease cascade in the regulation of neurotrophins in AD and, together with the findings described above, supports a role for neuroserpin at different stages in the development of AD.

### Role of neuroserpin in cancer

Neuroserpin has also been involved in different types of cancer. In prostate cancer, neuroserpin levels were upregulated, particularly in high-grade tumours, and was associated with shorter survival times [142]. In gastric cancer, a screening for targets of the oncogenic microRNA miR-21 identified *SERPINI1* as a target gene, and neuroserpin overexpression induced G1/S arrest and decreased the growth of MKN28 cells, suggesting a potential tumour suppressive function in this type of cancer [143]. A role for neuroserpin in promoting epithelial-mesenchymal transition in colorectal tumour cells has also been suggested, based on microarray analysis of cell lines and the effects of siRNA knock-down of *SERPINI1* gene in one of the cell lines [144]. A recent study of methylated gene profiles in bladder cancer has revealed that the *SERPINI1* gene is differentially methylated when comparing tumour and normal tissues [145]. In the nervous system, a study based on the analysis of single nucleotide polymorphisms (SNPs) in gliomas proposed a role for the *SERPINI1* gene, which appeared to be associated with glioblastoma, the most aggressive type of glioma [146]. In an elegant study, Valiente and colleagues demonstrated that brain metastatic cells from lung and breast cancers expressed high levels of anti-plasminogen activator serpins, including neuroserpin, which, by preventing the generation of plasmin, decreased the cleavage of FasL and L1CAM, thereby favouring brain metastasis in these types of cancer [98]. Also, in a whole-exome sequencing study, *SERPINI1* has been found as the most frequently mutated gene in brain metastasis [147], adding further evidence for a role of neuroserpin in this process.

## Neuroserpin alleles in population databases: functional and pathological implications

As described above, six pathogenic missense variants of *SERPINI1* have been associated to FENIB, and different studies support a role for neuroserpin in other types of dementia. This has led to the inclusion of *SERPINI1* in several genetic screenings to identify novel pathological variants [148, 149]. Next-generation sequencing of an early-onset dementia cohort of 246 patients has uncovered a missense variation in neuroserpin (Asp157Ser) in a patient diagnosed for Alzheimer’s disease [150]. The possibility of finding novel pathological variants by expanding the search has led us to interrogate public databases. ClinVar, a public archive linking human genomic variation to their clinical phenotype, presently annotates other 61 missense variations of *SERPINI1* encoding neuroserpin variants, 14 classified as benign or likely-benign, and 47 with uncertain clinical significance. The gnomAD database (versions v2.1/v3.1), which includes exome and genome sequencing data from approximately 200,000 unrelated individuals, collected as part of various disease-specific or population genetic studies, presently annotates 232 missense variations in *SERPINI1*, among which 220 have an allelic frequency < 5 × 10^5. Taken together, the new missense single nucleotide variations (SNVs) annotated in ClinVar and/or gnomAD encode for 220 unique amino acid substitutions in neuroserpin. As an initial step to estimate the pathogenic potential of these coding variants, we have applied two pathogenicity predictors: REVEL and Polyphen-2. REVEL is an ensemble method based on a combination of previously developed tools [151] and has been shown to achieve the best performance in discriminating benign *versus* pathogenic amino acid variations of α_1_-antitrypsin, the archetypal member of the serpin superfamily [152].

The amino acid variations with the highest predicted pathogenic potential according to REVEL scores (> 0.75) are listed in Table 2, along with scores assigned by Polyphen-2, a more widely used predictive tool [153]. These variations are widely distributed in neuroserpin’s structure, but 25% of the potentially pathogenic ones are located in β-sheet A, a critical region for serpin conformational changes. A second hotspot is located to helix D (16% of the mutations reported in Table 2), one of the helices lying in the protein’s core, where one of the FENIB mutations (Ser52Arg) was found. Many of the mutations in Table 2 introduce charged or bulky residues, likely causing significant protein destabilisation. Only one rather conservative mutation (Ala352Val) is located in the RCL, a motif that is central for neuroserpin’s activity. Experimental studies in vitro and in cell culture models will be required to assess the molecular behaviour of these neuroserpin variants with regards to intracellular polymerisation and anti-protease inhibitory activity. Notably, several loss-of-function variants of *SERPINI1* are also annotated in variation databases. Although these are not associated with the aggregation mechanisms underlying FENIB, their pathogenic potential in altering the extracellular functions of neuroserpin also deserves further investigation in vitro and/or in cellular and animal models of disease.

**Table 2:** List of SERPINI1 amino acid substitutions annotated in ClinVar and gnomAD (v2.1 and v3.1), achieving REVEL scores > 0.75

ClinVar				
Nucleotide change (ref. NM_005025.4)	Protein change	Clinical significance	REVEL score (> 0.750)	Polyphen-2 score
*c.1175G > A*	*p.Gly392Glu*	*Pathogenic*	*0.981*	*1.000*
*c.1174G > A*	*p.Gly392Arg*	*Pathogenic*	*0.976*	*1.000*
*c.145T > C*	*p.Ser49Pro*	*Pathogenic*	*0.965*	*1.000*
c.1139T > C	p.Ile380Thr	Uncertain	0.928	0.999
c.770T > C	p.Leu257Pro	Uncertain	0.909	1.000
c.526A > G	p.Thr176Ala*	Uncertain	0.908	1.000
*c.1013A > G*	*p.His338Arg*	*Pathogenic*	*0.899*	*0.999*
*c.154A > C*	*p.Ser52Arg*	*Pathogenic*	*0.858*	*1.000*
c.332C > T	p.Ser111Phe*	Uncertain	0.789	1.000
c.456T > G	p.Asn152Lys	Uncertain	0.779	1.000
c.959C > G	p.Ala320Gly	Uncertain	0.775	0.999
c.203C > A	p.Thr68Asn	Uncertain	0.761	0.997
c.166G > T	p.Ala56Ser*	Uncertain	0.752	1.000

gnomAD v2.1/v3.1				
Nucleotide change (ref. NM_005025.4)	Protein change	Allele counts	REVEL score (> 0.750)	Polyphen-2 score
c.149C > A	p.Pro50Gln	1	0.964	1.000
c.326C > A	p.Ala109Asp	2	0.960	1.000
c.920T > C	p.Leu307Ser	2	0.959	1.000
c.394T > C	p.Phe132Leu	1	0.953	1.000
c.647T > C	p.Met216Thr	1	0.942	0.999
c.1030G > T	p.Val344Phe	5	0.940	1.000
c.554A > G	p.Tyr185Cys	2	0.935	1.000
c.188G > T	p.Gly63Val	1	0.932	1.000
c.878C > T	p.Pro293Leu	1	0.928	0.996
c.595A > T	p.Thr199Ser	1	0.926	1.000
c.646A > G	p.Met216Val	1	0.920	0.993
c.172G > A	p.Gly58Arg	1	0.916	1.000
c.133A > G	p.Asn45Asp	1	0.912	1.000
c.526A > G	p.Thr176Ala*	3	0.908	1.000
c.187G > A	p.Gly63Arg	1	0.905	1.000
c.1177C > G	p.Arg393Gly	1	0.905	1.000
c.596C > T	p.Thr199Ile	3	0.902	1.000
c.1031T > C	p.Val344Ala	1	0.889	0.996
c.1033A > C	p.Asn345His	2	0.870	1.000
c.173G > A	p.Gly58Glu	2	0.868	1.000
c.551T > A	p.Val184Asp	3	0.863	0.999
c.935T > A	p.Ile312Lys	1	0.846	0.986
c.317T > A	p.Met106Lys	2	0.842	**0.349**
c.965T > G	p.Leu322Trp	2	0.832	0.995
c.882G > T	p.Arg294Ser	2	0.828	1.000
c.92T > C	p.Val31Ala	1	0.826	0.979
c.1055C > T	p.Ala352Val	1	0.809	1.000
c.1178G > A	p.Arg393Gln	1	0.809	1.000
c.184C > T	p.Leu62Phe	1	0.796	1.000
c.330T > A	p.Asn110Lys	1	0.796	1.000
c.866A > G	p.Glu289Gly	1	0.794	1.000
c.332C > T	p.Ser111Phe*	7	0.789	1.000
c.167C > G	p.Ala56Gly	1	0.781	0.999
c.769C > G	p.Leu257Val	1	0.773	0.995
c.623A > T	p.Asp208Val	1	0.762	1.000
c.344A > G	p.Gln115Arg	1	0.761	0.998
c.166G > T	p.Ala56Ser*	1	0.752	1.000

Databases were last accessed on 19 December 2020. Clinical significance refers to the pathogenicity prediction reported in ClinVar. Published mutations are highlighted in italics. A star (*) indicates substitutions annotated in both databases. Allele counts refer to total number of alleles in gnomAD (v2.1 or v3.1) datasets. The last column displays Polyphen-2 (HDIV) scores that classify almost all variants as probably damaging (> 0.908). Web resources:

ClinVar: https://www.ncbi.nlm.nih.gov/clinvar/

gnomAD (Genome Aggregation Database): https://gnomad.broadinstitute.org/

REVEL: https://sites.google.com/site/revelgenomics/

PolyPhen-2 (Polymorphism Phenotyping v2): http://genetics.bwh.harvard.edu/pph2/

## Conclusions and future perspectives

Most of the research dealing with neuroserpin has focused in two areas of interest: its roles in the physiology and pathologies of the central nervous system and the structural bases of its multiple conformational states. As for other serpins, these two aspects are intimately related for neuroserpin, since the structural changes described here are at the basis of neuroserpin’s anti-protease activity and the molecular mechanism of FENIB. In the context of the serpin superfamily, neuroserpin has attained a relevant model role along with α_1_-antitrypsin, due to the striking correlation between the destabilising effects of polymerogenic mutations causing FENIB and their clinical phenotype, and thanks to a solid body of studies dealing with the alternative conformations of neuroserpin and its polymerisation mechanism. FENIB, like all other serpinopathies, is currently an incurable disease. Different approaches have been tried for developing antipolymerisation molecules to treat α_1_-antitrypsin deficiency, the most common serpinopathy, including chemical chaperones, RCL-derived peptides and small molecules, but none of them has progressed to clinical application so far [2]. While the most common target for polymerisation inhibitors has been β-sheet A, a new promising molecule against α_1_-antitrypsin that binds a cryptic pocket in β-sheet B has been reported recently [154]. This region was already described as a possible binding site for small molecules against PAI-1 [155]. These findings point to stabilisation of the region between β-sheets B and C as a novel therapeutic option since it correlates with a decreased polymerisation rate. These promising results with α_1_-antitrypsin encourage further research on neuroserpin and FENIB, for which the only treatments available at this time are those aimed to relief the clinical symptoms.

Several aspects of the physiological and pathological functions of neuroserpin deserve to be further investigated, including novel functions in the CNS as well as in the immune system. It is particularly important to uncover new inhibitory and non-inhibitory mechanisms of action of neuroserpin and to exploit its possible therapeutical potential. The neuroprotective effect of neuroserpin administration into the neocortex and hippocampus following ischemic stroke and seizures, respectively [79, 85], encourages the development of novel strategies to deliver this serpin at the site of pathology. In this respect, adenoviral-mediated expression of neuroserpin in the brain has been shown to affect synaptic plasticity in both the hippocampus and the visual cortex [73, 81], proving the efficacy of this strategy and encouraging its optimisation for therapy. Moreover, the data on neuroserpin’s function in the immune system and in the vasculature published in the last years strongly support future studies about neuroserpin’s activity outside the CNS and promote novel therapies based on neuroserpin, as shown by the finding that peptides derived from its RCL exert immunomodulatory effects [156].

Finally, the finding in population databases of neuroserpin variants predicted to be deleterious, albeit at low frequency, together with the fact that late onset FENIB is probably diagnosed under the umbrella of ‘senile dementia’, supports the notion that the occurrence of FENIB is probably underestimated. In this scenario, the assessment of novel neuroserpin variants identified in cohorts of early-onset dementia or population screenings and the prediction of their pathological impact, which we have introduced here, will surely foster new perspectives.

## Abbreviations

BBB: Blood–brain barrier
BDNF: Brain-derived neurotrophic factor
CCL21: C–C motif ligand 21
CD: Circular dichroism
CNS: Central nervous system
EOR: Endoplasmic reticulum overload response
ER: Endoplasmic reticulum
ERAD: Endoplasmic reticulum associated degradation
FENIB: Familial encephalopathy with neuroserpin inclusion bodies
LRP: Low-density lipoprotein receptor-related protein
MCAO: Middle cerebral artery occlusion
NFκB: Nuclear factor κ-light-chain-enhancer of activated B cells
NGF: Nerve growth factor
RCL: Reactive centre loop
tPA: Tissue-type plasminogen activator
uPA: Urokinase-type plasminogen activator
UPR: Unfolded protein response

## References

[1] Lucas A, Yaron JR, Zhang L, Ambadapadi S (2018). Overview of serpins and their roles in biological systems. Methods Mol Biol Clifton NJ.

[2] Gooptu B, Lomas DA (2009). Conformational pathology of the serpins: themes, variations, and therapeutic strategies. Annu Rev Biochem.

[3] Gooptu B, Dickens JA, Lomas DA (2014). The molecular and cellular pathology of α_1_-antitrypsin deficiency. Trends Mol Med.

[4] Roussel BD, Irving JA, Ekeowa UI (2011). Unravelling the twists and turns of the serpinopathies. FEBS J.

[5] Lee TW, Tsang VWK, Loef EJ, Birch NP (2017). Physiological and pathological functions of neuroserpin: regulation of cellular responses through multiple mechanisms. Semin Cell Dev Biol.

[6] Stoeckli ET, Lemkin PF, Kuhn TB (1989). Identification of proteins secreted from axons of embryonic dorsal-root-ganglia neurons. Eur J Biochem.

[7] Osterwalder T, Contartese J, Stoeckli ET (1996). Neuroserpin, an axonally secreted serine protease inhibitor. EMBO J.

[8] Silverman GA, Bird PI, Carrell RW (2001). The serpins are an expanding superfamily of structurally similar but functionally diverse proteins. Evolution, mechanism of inhibition, novel functions, and a revised nomenclature. J Biol Chem.

[9] Schrimpf SP, Bleiker AJ, Brecevic L (1997). Human neuroserpin (PI12): cDNA cloning and chromosomal localization to 3q26. Genomics.

[10] Yazaki M, Liepnieks JJ, Murrell JR (2001). Biochemical characterization of a neuroserpin variant associated with hereditary dementia. Am J Pathol.

[11] Krueger SR, Ghisu GP, Cinelli P (1997). Expression of neuroserpin, an inhibitor of tissue plasminogen activator, in the developing and adult nervous system of the mouse. J Neurosci Off J Soc Neurosci.

[12] Hastings GA, Coleman TA, Haudenschild CC (1997). Neuroserpin, a brain-associated inhibitor of tissue plasminogen activator is localized primarily in neurons. J Biol Chem.

[13] Adorjan I, Tyler T, Bhaduri A (2019). Neuroserpin expression during human brain development and in adult brain revealed by immunohistochemistry and single cell RNA sequencing. J Anat.

[14] de Groot DM, Pol C, Martens GJM (2005). Comparative analysis and expression of neuroserpin in *Xenopus laevis*. Neuroendocrinology.

[15] Kennedy SA, van Diepen AC, van den Hurk CM (2007). Expression of the serine protease inhibitor neuroserpin in cells of the human myeloid lineage. Thromb Haemost.

[16] Lorenz N, Loef EJ, Verdon DJ (2015). Human T cell activation induces synaptic translocation and alters expression of the serine protease inhibitor neuroserpin and its target protease. J Leukoc Biol.

[17] Hermann M, Reumann R, Schostak K (2020). Deficits in developmental neurogenesis and dendritic spine maturation in mice lacking the serine protease inhibitor neuroserpin. Mol Cell Neurosci.

[18] Kement D, Reumann R, Schostak K (2021). Neuroserpin is strongly expressed in the developing and adult mouse neocortex but its absence does not perturb cortical lamination and synaptic proteome. Front Neuroanat.

[19] Belorgey D, Crowther DC, Mahadeva R, Lomas DA (2002). Mutant neuroserpin (S49P) that causes familial encephalopathy with neuroserpin inclusion bodies is a poor proteinase inhibitor and readily forms polymers in vitro. J Biol Chem.

[20] Belorgey D, Sharp LK, Crowther DC (2004). Neuroserpin Portland (Ser52Arg) is trapped as an inactive intermediate that rapidly forms polymers: implications for the epilepsy seen in the dementia FENIB. Eur J Biochem.

[21] Onda M, Belorgey D, Sharp LK, Lomas DA (2005). Latent S49P neuroserpin forms polymers in the dementia familial encephalopathy with neuroserpin inclusion bodies. J Biol Chem.

[22] Briand C, Kozlov SV, Sonderegger P, Grütter MG (2001). Crystal structure of neuroserpin: a neuronal serpin involved in a conformational disease. FEBS Lett.

[23] Ricagno S, Caccia S, Sorrentino G (2009). Human neuroserpin: structure and time-dependent inhibition. J Mol Biol.

[24] Takehara S, Onda M, Zhang J (2009). The 2.1-Å crystal structure of native neuroserpin reveals unique structural elements that contribute to conformational instability. J Mol Biol.

[25] Zhang Q, Buckle AM, Law RHP (2007). The N terminus of the serpin, tengpin, functions to trap the metastable native state. EMBO Rep.

[26] Yang L, Irving JA, Dai W (2018). Probing the folding pathway of a consensus serpin using single tryptophan mutants. Sci Rep.

[27] Ali MF, Kaushik A, Kapil C (2017). A hydrophobic patch surrounding Trp154 in human neuroserpin controls the helix F dynamics with implications in inhibition and aggregation. Sci Rep.

[28] Noto R, Santangelo MG, Levantino M (2015). Functional and dysfunctional conformers of human neuroserpin characterized by optical spectroscopies and Molecular Dynamics. Biochim Biophys Acta.

[29] Sarkar A, Zhou C, Meklemburg R, Wintrode PL (2011). Local conformational flexibility provides a basis for facile polymer formation in human neuroserpin. Biophys J.

[30] Noto R, Randazzo L, Raccosta S (2015). The stability and activity of human neuroserpin are modulated by a salt bridge that stabilises the reactive centre loop. Sci Rep.

[31] Elliott PR, Pei XY, Dafforn TR, Lomas DA (2000). Topography of a 2.0 A structure of alpha1-antitrypsin reveals targets for rational drug design to prevent conformational disease. Protein Sci Publ Protein Soc.

[32] Lawrence DA, Ginsburg D, Day DE (1995). Serpin-protease complexes are trapped as stable acyl-enzyme intermediates. J Biol Chem.

[33] Whisstock JC, Bottomley SP (2006). Molecular gymnastics: serpin structure, folding and misfolding. Curr Opin Struct Biol.

[34] Huntington JA, Read RJ, Carrell RW (2000). Structure of a serpin–protease complex shows inhibition by deformation. Nature.

[35] Nykjaer A, Petersen CM, Møller B (1992). Purified alpha 2-macroglobulin receptor/LDL receptor-related protein binds urokinase.plasminogen activator inhibitor type-1 complex. Evidence that the alpha 2-macroglobulin receptor mediates cellular degradation of urokinase receptor-bound complexes. J Biol Chem.

[36] Bu G, Maksymovitch EA, Schwartz AL (1993). Receptor-mediated endocytosis of tissue-type plasminogen activator by low density lipoprotein receptor-related protein on human hepatoma HepG2 cells. J Biol Chem.

[37] Makarova A, Mikhailenko I, Bugge TH (2003). The low density lipoprotein receptor-related protein modulates protease activity in the brain by mediating the cellular internalization of both neuroserpin and neuroserpin-tissue-type plasminogen activator complexes. J Biol Chem.

[38] Barker-Carlson K, Lawrence DA, Schwartz BS (2002). Acyl-enzyme complexes between tissue-type plasminogen activator and neuroserpin are short-lived in vitro. J Biol Chem.

[39] Lee TW, Yang AS-P, Brittain T, Birch NP (2015). An analysis approach to identify specific functional sites in orthologous proteins using sequence and structural information: application to neuroserpin reveals regions that differentially regulate inhibitory activity: identification of specific functional sites. Proteins.

[40] Carlson K-SB, Nguyen L, Schwartz K (2016). Neuroserpin differentiates between forms of tissue type plasminogen activator via pH dependent deacylation. Front Cell Neurosci.

[41] Visentin C, Broggini L, Sala BM (2020). Glycosylation tunes neuroserpin physiological and pathological properties. Int J Mol Sci.

[42] Stout TJ, Graham H, Buckley DI, Matthews DJ (2000). Structures of active and latent PAI-1: a possible stabilizing role for chloride ions ^‡^. Biochemistry.

[43] Ricagno S, Pezzullo M, Barbiroli A (2010). Two latent and two hyperstable polymeric forms of human neuroserpin. Biophys J.

[44] Lomas DA, Belorgey D, Mallya M (2004). Polymerisation underlies alpha1-antitrypsin deficiency, dementia and other serpinopathies. Front Biosci J Virtual Libr.

[45] Belorgey D, Irving JA, Ekeowa UI (2011). Characterisation of serpin polymers in vitro and in vivo. Methods San Diego Calif.

[46] Lomas DA, Evans DL, Finch JT, Carrell RW (1992). The mechanism of Z alpha 1-antitrypsin accumulation in the liver. Nature.

[47] Yamasaki M, Li W, Johnson DJD, Huntington JA (2008). Crystal structure of a stable dimer reveals the molecular basis of serpin polymerization. Nature.

[48] Yamasaki M, Sendall TJ, Pearce MC (2011). Molecular basis of α1-antitrypsin deficiency revealed by the structure of a domain-swapped trimer. EMBO Rep.

[49] Faull SV, Elliston ELK, Gooptu B (2020). The structural basis for Z α1-antitrypsin polymerization in the liver. Sci Adv.

[50] Raccosta S, Librizzi F, Jagger AM (2021). Scaling concepts in serpin polymer physics. Materials.

[51] Noto R, Santangelo MG, Ricagno S (2012). The tempered polymerization of human neuroserpin. PLoS One.

[52] Davis RL, Shrimpton AE, Holohan PD (1999). Familial dementia caused by polymerization of mutant neuroserpin. Nature.

[53] Saga G, Sessa F, Barbiroli A (2016). Embelin binds to human neuroserpin and impairs its polymerisation. Sci Rep.

[54] Miranda E, Römisch K, Lomas DA (2004). Mutants of neuroserpin that cause dementia accumulate as polymers within the endoplasmic reticulum. J Biol Chem.

[55] Takehara S, Zhang J, Yang X (2010). Refolding and polymerization pathways of neuroserpin. J Mol Biol.

[56] Santangelo MG, Noto R, Levantino M (2012). On the molecular structure of human neuroserpin polymers. Proteins.

[57] Ali MF, Kaushik A, Gupta D (2020). Changes in strand 6B and helix B during neuroserpin inhibition: implication in severity of clinical phenotype. Biochim Biophys Acta Proteins Proteom.

[58] Belorgey D, Hägglöf P, Onda M, Lomas DA (2010). pH-dependent stability of neuroserpin is mediated by histidines 119 and 138; implications for the control of β-sheet A and polymerization: pH-dependent stability of neuroserpin. Protein Sci.

[59] Sharp LK, Mallya M, Kinghorn KJ (2006). Sugar and alcohol molecules provide a therapeutic strategy for the serpinopathies that cause dementia and cirrhosis. FEBS J.

[60] Kinghorn KJ, Crowther DC, Sharp LK (2006). Neuroserpin binds Aβ and is a neuroprotective component of amyloid plaques in Alzheimer disease. J Biol Chem.

[61] Chiou A, Hägglöf P, Orte A (2009). Probing neuroserpin polymerization and interaction with amyloid-beta peptides using single molecule fluorescence. Biophys J.

[62] Andersen CB, Yagi H, Manno M (2009). Branching in amyloid fibril growth. Biophys J.

[63] Budrikis Z, Costantini G, La Porta CAM, Zapperi S (2014). Protein accumulation in the endoplasmic reticulum as a non-equilibrium phase transition. Nat Commun.

[64] Giampietro C, Lionetti MC, Costantini G (2017). Cholesterol impairment contributes to neuroserpin aggregation. Sci Rep.

[65] Visentin C, Musso L, Broggini L (2020). Embelin as lead compound for new neuroserpin polymerization inhibitors. Life Basel Switz.

[66] Miranda E, MacLeod I, Davies MJ (2008). The intracellular accumulation of polymeric neuroserpin explains the severity of the dementia FENIB. Hum Mol Genet.

[67] Davies MJ, Miranda E, Roussel BD (2009). Neuroserpin polymers activate NF-κB by a calcium signaling pathway that is independent of the unfolded protein response. J Biol Chem.

[68] Guadagno NA, Moriconi C, Licursi V (2017). Neuroserpin polymers cause oxidative stress in a neuronal model of the dementia FENIB. Neurobiol Dis.

[69] Schipanski A, Oberhauser F, Neumann M (2014). Lectin OS-9 delivers mutant neuroserpin to endoplasmic reticulum associated degradation in familial encephalopathy with neuroserpin inclusion bodies. Neurobiol Aging.

[70] Moriconi C, Ordoñez A, Lupo G (2015). Interactions between N-linked glycosylation and polymerisation of neuroserpin within the endoplasmic reticulum. FEBS J.

[71] Osterwalder T, Cinelli P, Baici A (1998). The axonally secreted serine proteinase inhibitor, neuroserpin, inhibits plasminogen activators and plasmin but not thrombin. J Biol Chem.

[72] Cinelli P, Madani R, Tsuzuki N (2001). Neuroserpin, a neuroprotective factor in focal ischemic stroke. Mol Cell Neurosci.

[73] Tsang VWK, Young D, During MJ, Birch NP (2014). AAV-mediated overexpression of neuroserpin in the hippocampus decreases PSD-95 expression but does not affect hippocampal-dependent learning and memory. PLoS One.

[74] Teesalu T, Kulla A, Simisker A (2004). Tissue plasminogen activator and neuroserpin are widely expressed in the human central nervous system. Thromb Haemost.

[75] Seeds NW, Basham ME, Haffke SP (1999). Neuronal migration is retarded in mice lacking the tissue plasminogen activator gene. Proc Natl Acad Sci USA.

[76] Huang YY, Bach ME, Lipp HP (1996). Mice lacking the gene encoding tissue-type plasminogen activator show a selective interference with late-phase long-term potentiation in both Schaffer collateral and mossy fiber pathways. Proc Natl Acad Sci USA.

[77] Lee SH, Ko HM, Kwon KJ (2014). tPA regulates neurite outgrowth by phosphorylation of LRP5/6 in neural progenitor cells. Mol Neurobiol.

[78] Wang YF, Tsirka SE, Strickland S (1998). Tissue plasminogen activator (tPA) increase neuronal damage after focal cerebral ischemia in wild-type and tPA-deficient mice. Nat Med.

[79] Yepes M, Sandkvist M, Coleman TA (2002). Regulation of seizure spreading by neuroserpin and tissue-type plasminogen activator is plasminogen-independent. J Clin Invest.

[80] Fredriksson L, Stevenson TK, Su EJ (2015). Identification of a neurovascular signaling pathway regulating seizures in mice. Ann Clin Transl Neurol.

[81] Bukhari N, Burman PN, Hussein A (2015). Unmasking proteolytic activity for adult visual cortex plasticity by the removal of lynx1. J Neurosci.

[82] Lorenz N, Loef EJ, Kelch ID (2016). Plasmin and regulators of plasmin activity control the migratory capacity and adhesion of human T cells and dendritic cells by regulating cleavage of the chemokine CCL21. Immunol Cell Biol.

[83] Loef EJ, Brooks AES, Lorenz N (2020). Neuroserpin regulates human T cell-T cell interactions and proliferation through inhibition of tissue plasminogen activator. J Leukoc Biol.

[84] Madani R, Kozlov S, Akhmedov A (2003). Impaired explorative behavior and neophobia in genetically modified mice lacking or overexpressing the extracellular serine protease inhibitor neuroserpin. Mol Cell Neurosci.

[85] Yepes M, Sandkvist M, Wong MKK (2000). Neuroserpin reduces cerebral infarct volume and protects neurons from ischemia-induced apoptosis. Blood.

[86] Ma J, Yu D, Tong Y, Mao M (2012). Effect of neuroserpin in a neonatal hypoxic-ischemic injury model ex vivo. Biol Res.

[87] Lebeurrier N, Liot G, Lopez-Atalaya JP (2005). The brain-specific tissue-type plasminogen activator inhibitor, neuroserpin, protects neurons against excitotoxicity both in vitro and in vivo. Mol Cell Neurosci.

[88] Wu J, Echeverry R, Guzman J, Yepes M (2010). Neuroserpin protects neurons from ischemia-induced plasmin-mediated cell death independently of tissue-type plasminogen activator inhibition. Am J Pathol.

[89] Gu RP, Fu LL, Jiang CH (2015). Retina is protected by neuroserpin from ischemic/reperfusion-induced injury independent of tissue-type plasminogen activator. PLoS One.

[90] Lee TW, Coates LC, Birch NP (2008). Neuroserpin regulates N-cadherin-mediated cell adhesion independently of its activity as an inhibitor of tissue plasminogen activator. J Neurosci Res.

[91] May P, Rohlmann A, Bock HH (2004). Neuronal LRP1 functionally associates with postsynaptic proteins and is required for normal motor function in mice. Mol Cell Biol.

[92] Narita M, Bu G, Holtzman DM, Schwartz AL (1997). The low-density lipoprotein receptor-related protein, a multifunctional apolipoprotein E receptor, modulates hippocampal neurite development. J Neurochem.

[93] Hill RM, Parmar PK, Coates LC (2000). Neuroserpin is expressed in the pituitary and adrenal glands and induces the extension of neurite-like processes in AtT-20 cells. Biochem J.

[94] Parmar PK, Coates LC, Pearson JF (2002). Neuroserpin regulates neurite outgrowth in nerve growth factor-treated PC12 cells. J Neurochem.

[95] Han S, Fei F, Sun S (2021). Increased anxiety was found in serpini1 knockout zebrafish larval. Biochem Biophys Res Commun.

[96] Borges VM, Lee TW, Christie DL, Birch NP (2010). Neuroserpin regulates the density of dendritic protrusions and dendritic spine shape in cultured hippocampal neurons. J Neurosci Res.

[97] Reumann R, Vierk R, Zhou L (2017). The serine protease inhibitor neuroserpin is required for normal synaptic plasticity and regulates learning and social behavior. Learn Mem Cold Spring Harb N.

[98] Valiente M, Obenauf AC, Jin X (2014). Serpins promote cancer cell survival and vascular co-option in brain metastasis. Cell.

[99] Rodríguez-González R, Agulla J, Pérez-Mato M (2011). Neuroprotective effect of neuroserpin in rat primary cortical cultures after oxygen and glucose deprivation and tPA. Neurochem Int.

[100] Munuswamy-Ramanujam G, Dai E, Liu L (2010). Neuroserpin, a thrombolytic serine protease inhibitor (serpin), blocks transplant vasculopathy with associated modification of T-helper cell subsets. Thromb Haemost.

[101] Davis RL, Holohan PD, Shrimpton AE (1999). Familial encephalopathy with neuroserpin inclusion bodies. Am J Pathol.

[102] Bradshaw CB, Davis RL, Shrimpton AE (2001). Cognitive deficits associated with a recently reported familial neurodegenerative disease: familial encephalopathy with neuroserpin inclusion bodies. Arch Neurol.

[103] Roussel BD, Lomas DA, Crowther DC (2016). Progressive myoclonus epilepsy associated with neuroserpin inclusion bodies (neuroserpinosis). Epileptic Disord.

[104] Davis RL, Shrimpton AE, Carrell RW (2002). Association between conformational mutations in neuroserpin and onset and severity of dementia. Lancet.

[105] Hagen MC, Murrell JR, Delisle M-B (2011). Encephalopathy with neuroserpin inclusion bodies presenting as progressive myoclonus epilepsy and associated with a novel mutation in the Proteinase Inhibitor 12 gene. Brain Pathol Zurich Switz.

[106] Laffranchi M, Berardelli R, Ronzoni R (2018). Heteropolymerization of α-1-antitrypsin mutants in cell models mimicking heterozygosity. Hum Mol Genet.

[107] Laffranchi M, Elliston ELK, Miranda E (2020). Intrahepatic heteropolymerization of M and Z alpha-1-antitrypsin. JCI Insight.

[108] Galliciotti G, Glatzel M, Kinter J (2007). Accumulation of mutant neuroserpin precedes development of clinical symptoms in familial encephalopathy with neuroserpin inclusion bodies. Am J Pathol.

[109] Takao M, Benson MD, Murrell JR (2000). Neuroserpin mutation S52R causes neuroserpin accumulation in neurons and is associated with progressive myoclonus epilepsy. J Neuropathol Exp Neurol.

[110] Gourfinkel-An I, Duyckaerts C, Camuzat A (2007). Clinical and neuropathologic study of a French family with a mutation in the neuroserpin gene. Neurology.

[111] Coutelier M, Andries S, Ghariani S (2008). Neuroserpin mutation causes electrical status epilepticus of slow-wave sleep. Neurology.

[112] Roussel BD, Kruppa AJ, Miranda E (2013). Endoplasmic reticulum dysfunction in neurological disease. Lancet Neurol.

[113] Takasawa A, Kato I, Takasawa K (2008). Mutation-, aging-, and gene dosage-dependent accumulation of neuroserpin (G392E) in endoplasmic reticula and lysosomes of neurons in transgenic mice. J Biol Chem.

[114] Kroeger H, Miranda E, MacLeod I (2009). Endoplasmic reticulum-associated degradation (ERAD) and autophagy cooperate to degrade polymerogenic mutant serpins. J Biol Chem.

[115] Ying Z, Wang H, Fan H, Wang G (2011). The endoplasmic reticulum (ER)-associated degradation system regulates aggregation and degradation of mutant neuroserpin. J Biol Chem.

[116] Lawless MW, Greene CM, Mulgrew A (1950). (2004) Activation of endoplasmic reticulum-specific stress responses associated with the conformational disease Z alpha 1-antitrypsin deficiency. J Immunol Baltim Md.

[117] Hidvegi T, Schmidt BZ, Hale P, Perlmutter DH (2005). Accumulation of mutant α_1_ -antitrypsin Z in the endoplasmic reticulum activates caspases-4 and -12, NFκB, and BAP31 but not the unfolded protein response. J Biol Chem.

[118] López-González I, Pérez-Mediavilla A, Zamarbide M (2016). Limited unfolded protein response and inflammation in neuroserpinopathy. J Neuropathol Exp Neurol.

[119] Pahl HL (1999). Signal transduction from the endoplasmic reticulum to the cell nucleus. Physiol Rev.

[120] Marcus NY, Blomenkamp K, Ahmad M, Teckman JH (2012). Oxidative stress contributes to liver damage in a murine model of alpha-1-antitrypsin deficiency. Exp Biol Med.

[121] Escribano A, Amor M, Pastor S (2015). Decreased glutathione and low catalase activity contribute to oxidative stress in children with α-1 antitrypsin deficiency: Table 1. Thorax.

[122] Zhang Z, Zhang L, Yepes M (2002). Adjuvant treatment with neuroserpin increases the therapeutic window for tissue-type plasminogen activator administration in a rat model of embolic stroke. Circulation.

[123] Gelderblom M, Neumann M, Ludewig P (2013). Deficiency in serine protease inhibitor neuroserpin exacerbates ischemic brain injury by increased postischemic inflammation. PLoS One.

[124] Yepes M, Sandkvist M, Moore EG (2003). Tissue-type plasminogen activator induces opening of the blood-brain barrier via the LDL receptor-related protein. J Clin Invest.

[125] Wang L, Zhang Y, Asakawa T (2015). Neuroprotective effect of neuroserpin in oxygen-glucose deprivation- and reoxygenation-treated rat astrocytes in vitro. PLoS One.

[126] Yang X, Asakawa T, Han S (2016). Neuroserpin protects rat neurons and microglia-mediated inflammatory response against oxygen-glucose deprivation- and reoxygenation treatments in an in vitro study. Cell Physiol Biochem Int J Exp Cell Physiol Biochem Pharmacol.

[127] Rodríguez-González R, Millán M, Sobrino T (2011). The natural tissue plasminogen activator inhibitor neuroserpin and acute ischaemic stroke outcome. Thromb Haemost.

[128] Rodríguez-González R, Sobrino T, Rodríguez-Yáñez M (2011). Association between neuroserpin and molecular markers of brain damage in patients with acute ischemic stroke. J Transl Med.

[129] Cai L, Zhou Y, Wang Z, Zhu Y (2020). Neuroserpin extends the time window of tPA thrombolysis in a rat model of middle cerebral artery occlusion. J Biochem Mol Toxicol.

[130] Hakak Y, Walker JR, Li C (2001). Genome-wide expression analysis reveals dysregulation of myelination-related genes in chronic schizophrenia. Proc Natl Acad Sci.

[131] Wen Z, Nguyen HN, Guo Z (2014). Synaptic dysregulation in a human iPS cell model of mental disorders. Nature.

[132] Vawter MP, Weickert CS, Ferran E (2004). Gene expression of metabolic enzymes and a protease inhibitor in the prefrontal cortex are decreased in schizophrenia. Neurochem Res.

[133] Han W, Dang R, Xu P (2019). Altered fibrinolytic system in rat models of depression and patients with first-episode depression. Neurobiol Stress.

[134] Fabbro S, Seeds NW (2009). Plasminogen activator activity is inhibited while neuroserpin is up-regulated in the Alzheimer disease brain. J Neurochem.

[135] Fabbro S, Schaller K, Seeds NW (2011). Amyloid-beta levels are significantly reduced and spatial memory defects are rescued in a novel neuroserpin-deficient Alzheimer’s disease transgenic mouse model: cognitive deficit rescued in novel transgenic mouse. J Neurochem.

[136] Nielsen HM, Minthon L, Londos E (2007). Plasma and CSF serpins in Alzheimer disease and dementia with Lewy bodies. Neurology.

[137] Barker R, Kehoe PG, Love S (2012). Activators and inhibitors of the plasminogen system in Alzheimer’s disease. J Cell Mol Med.

[138] Hanzel CE, Iulita MF, Eyjolfsdottir H (2014). Analysis of matrix metallo-proteases and the plasminogen system in mild cognitive impairment and Alzheimer’s disease cerebrospinal fluid. J Alzheimers Dis.

[139] Subhadra B, Schaller K, Seeds NW (2013). Neuroserpin up-regulation in the Alzheimer’s disease brain is associated with elevated thyroid hormone receptor-β1 and HuD expression. Neurochem Int.

[140] Bruno MA, Leon WC, Fragoso G (2009). Amyloid beta-induced nerve growth factor dysmetabolism in Alzheimer disease. J Neuropathol Exp Neurol.

[141] Iulita MF, Bistué Millón MB, Pentz R (2017). Differential deregulation of NGF and BDNF neurotrophins in a transgenic rat model of Alzheimer’s disease. Neurobiol Dis.

[142] Hasumi H, Ishiguro H, Nakamura M (2005). Neuroserpin (PI-12) is upregulated in high-grade prostate cancer and is associated with survival. Int J Cancer.

[143] Yamanaka S, Olaru AV, An F (2012). MicroRNA-21 inhibits Serpini1, a gene with novel tumour suppressive effects in gastric cancer. Dig Liver Dis.

[144] Matsuda Y, Miura K, Yamane J (2016). SERPINI1 regulates epithelial-mesenchymal transition in an orthotopic implantation model of colorectal cancer. Cancer Sci.

[145] Yang Z, Liu A, Xiong Q (2019). Prognostic value of differentially methylated gene profiles in bladder cancer. J Cell Physiol.

[146] Rajaraman P, Brenner AV, Butler MA (2009). Common variation in genes related to innate immunity and risk of adult glioma. Cancer Epidemiol Biomarkers Prev.

[147] Richichi C, Fornasari L, Melloni GEM (2017). Mutations targeting the coagulation pathway are enriched in brain metastases. Sci Rep.

[148] Beck J, Pittman A, Adamson G (2014). Validation of next-generation sequencing technologies in genetic diagnosis of dementia. Neurobiol Aging.

[149] Bonvicini C, Scassellati C, Benussi L (2019). Next generation sequencing analysis in early onset dementia patients. J Alzheimers Dis.

[150] Bartoletti-Stella A, Baiardi S, Stanzani-Maserati M (2018). Identification of rare genetic variants in Italian patients with dementia by targeted gene sequencing. Neurobiol Aging.

[151] Ioannidis NM, Rothstein JH, Pejaver V (2016). REVEL: an ensemble method for predicting the pathogenicity of rare missense variants. Am J Hum Genet.

[152] Giacopuzzi E, Laffranchi M, Berardelli R (2018). Real-world clinical applicability of pathogenicity predictors assessed on SERPINA1 mutations in alpha-1-antitrypsin deficiency. Hum Mutat.

[153] Adzhubei IA, Schmidt S, Peshkin L (2010). A method and server for predicting damaging missense mutations. Nat Methods.

[154] Lomas DA, Irving JA, Arico-Muendel C (2021). Development of a small molecule that corrects misfolding and increases secretion of Z α1 -antitrypsin. EMBO Mol Med.

[155] Li S-H, Reinke AA, Sanders KL (2013). Mechanistic characterization and crystal structure of a small molecule inactivator bound to plasminogen activator inhibitor-1. Proc Natl Acad Sci.

[156] Ambadapadi S, Munuswamy-Ramanujam G, Zheng D (2016). Reactive center loop (RCL) peptides derived from serpins display independent coagulation and immune modulating activities. J Biol Chem.

[157] Osterwalder T (2004). Drosophila serpin 4 functions as a neuroserpin-like inhibitor of subtilisin-like proprotein convertases. J Neurosci.

[158] Simonin Y, Charron Y, Sonderegger P (2006). An inhibitor of serine proteases, neuroserpin, acts as a neuroprotective agent in a mouse model of neurodegenerative disease. J Neurosci.

[159] Ingwersen T, Linnenberg C, D’Acunto E (2021). G392E neuroserpin causing the dementia FENIB is secreted from cells but is not synaptotoxic. Sci Rep.

